# Emergency Care Gap in Brazil: Geographical Accessibility as a Proxy of Response Capacity to Tackle COVID-19

**DOI:** 10.3389/fpubh.2021.740284

**Published:** 2021-11-16

**Authors:** Lincoln Luís Silva, Amanda de Carvalho Dutra, Luciano de Andrade, Pedro Henrique Iora, Guilherme Luiz Rodrigues Ramajo, Iago Amado Peres Gualda, João Felipe Hermann Costa Scheidt, Pedro Vasconcelos Maia do Amaral, Thiago Augusto Hernandes Rocha, Catherine Ann Staton, João Ricardo Nickenig Vissoci, Rosilene Fressatti Cardoso

**Affiliations:** ^1^Graduate Program in Biosciences and Physiopathology, State University of Maringá, Paraná, Brazil; ^2^Graduate Program in Health Sciences, State University of Maringá, Paraná, Brazil; ^3^Department of Medicine, State University of Maringá, Paraná, Brazil; ^4^Faculty of Economic Sciences, Federal University of Minas Gerais, Belo Horizonte, Brazil; ^5^Global Emergency Medicine Innovation and Implementation (GEMINI), Division of Emergency Medicine, Duke Global Health Institute, Durham, NC, United States

**Keywords:** COVID-19, epidemiology, spatial analysis, public health, health services accessibility

## Abstract

**Background:** The new coronavirus disease (COVID-19) has claimed thousands of lives worldwide and disrupted the health system in many countries. As the national emergency care capacity is a crucial part of the COVID-19 response, we evaluated the Brazilian Health Care System response preparedness against the COVID-19 pandemic.

**Methods:** A retrospective and ecological study was performed with data retrieved from the Brazilian Information Technology Department of the Public Health Care System. The numbers of intensive care (ICU) and hospital beds, general or intensivist physicians, nurses, nursing technicians, physiotherapists, and ventilators from each health region were extracted. Beds per health professionals and ventilators per population rates were assessed. A health service accessibility index was created using a two-step floating catchment area (2SFCA). A spatial analysis using Getis-Ord Gi^*^ was performed to identify areas lacking access to high-complexity centers (HCC).

**Results:** As of February 2020, Brazil had 35,682 ICU beds, 426,388 hospital beds, and 65,411 ventilators. In addition, 17,240 new ICU beds were created in June 2020. The South and Southeast regions have the highest rates of professionals and infrastructure to attend patients with COVID-19 compared with the northern region. The north region has the lowest accessibility to ICUs.

**Conclusions:** The Brazilian Health Care System is unevenly distributed across the country. The inequitable distribution of health facilities, equipment, and human resources led to inadequate preparedness to manage the COVID-19 pandemic. In addition, the ineffectiveness of public measures of the municipal and federal administrations aggravated the pandemic in Brazil.

## Introduction

In December 2019, the novel severe acute respiratory syndrome coronavirus-2 (SARS-CoV-2) was identified as the causative agent of coronavirus 2019 (COVID-19) (1). The WHO declared COVID-19 a pandemic disease by the end of January 2020 (1). As of July 6, 2021, COVID-19 infected more than 183 million people and resulted in almost 4 million deaths globally (2). However, Brazil, where the virus reached much later than other countries, is one of the most affected countries with 19.1 million confirmed cases and 533,000 deaths reported so far (2). This case burden put the Brazilian health service at risk of total collapse (3).

To provide necessary health system resources to adequately overcome COVID-19, a strong coordinated emergency response with adequate facilities, personnel, and equipment is required (4). Resource constraints, including a lack of intensive care, inequitable distribution of hospital beds, and inadequate numbers of ventilators, compromise the capacity of the health system to care for patients with COVID-19 (5). In addition, the Brazilian government has faced several challenges while seeking the funds for the public Unified Health System (SUS) over the last decade (6). The lack of funds to support the public health system has led to a deterioration of health capacity among smaller municipalities and exacerbating inequalities (6).

The Unified Health System (Sistema Único de Saúde, SUS) of Brazil is the Brazil Health System. Brazil is the only country with a population of more than 200 million people to have a universal health care system, where almost 75% of the population rely on the service (7). SUS is maintained by public power, with supplementary participation of private initiatives, which guarantees free health care for all citizens, which is based primarily on equity, universality, and integrality (7, 8). However, as Brazil is a country of continental dimensions (8), geographic distribution of hospitals and physicians does not necessarily reach all Brazilians, turning healthcare accessibility into a severe problem. Many studies have recently used the Geographic Information System (GIS) to verify the accessibility to healthcare or to identify poorly served areas (9–11). The two-step floating catchment area (2SFCA) is, by far, the most used GIS technique to verify the accessibility to care, calculating the ratio of suppliers to the population within a service area centered at the location of a supplier (12). Such a method provides useful information on resources management by addressing the necessary support to the most deprived location of the country.

Globally, healthcare systems have collapsed due to COVID-19 (13). Hospital administrators, governments, policy-makers, and researchers must prepare for an immense surge of patients overloading the healthcare system. Thus, to verify the Brazilian healthcare preparedness in pandemic times, this study aims to analyze the geospatial distribution of healthcare resources (intensive care units and hospital beds, ventilators, and hospitals) and the access to establishments with intensive care units (ICU).

## Materials and Methods

### Study Design

This is an ecological, observational, and cross-sectional study based on secondary data. The aim was to evaluate the Brazilian health system infrastructure based on the amount of health resources (ICU and hospital beds, health professionals, and the accessibility to ICU beds) to verify the Brazilian preparedness for the COVID-19 pandemic.

### The Brazilian Health System

The SUS is the Brazilian public health system created by the Brazilian Constitution of 1988 (6, 14). SUS provides the Brazilian people the right to universal and free health care, from primary care to highly complex procedures. In addition, to reduce local and regional inequality, Brazil was divided into 438 health regions to correct these distortions among the States in order to optimize the distribution of resources and efficiency in the health care network (14). Health regions are delimited by an aggregate of several municipalities that ensure comprehensive healthcare assistance to the population within the region. Larger municipalities are responsible to provide any scale of care complexity and handle other administrative and technological complexities that can be used by citizens of other cities in the same region. Therefore, the citizens of a small town requiring a more complex service will resort to the services offered by a larger city within a given healthcare region (15).

### Setting

#### Demographic and Socioeconomic Information

**Figure 1:**
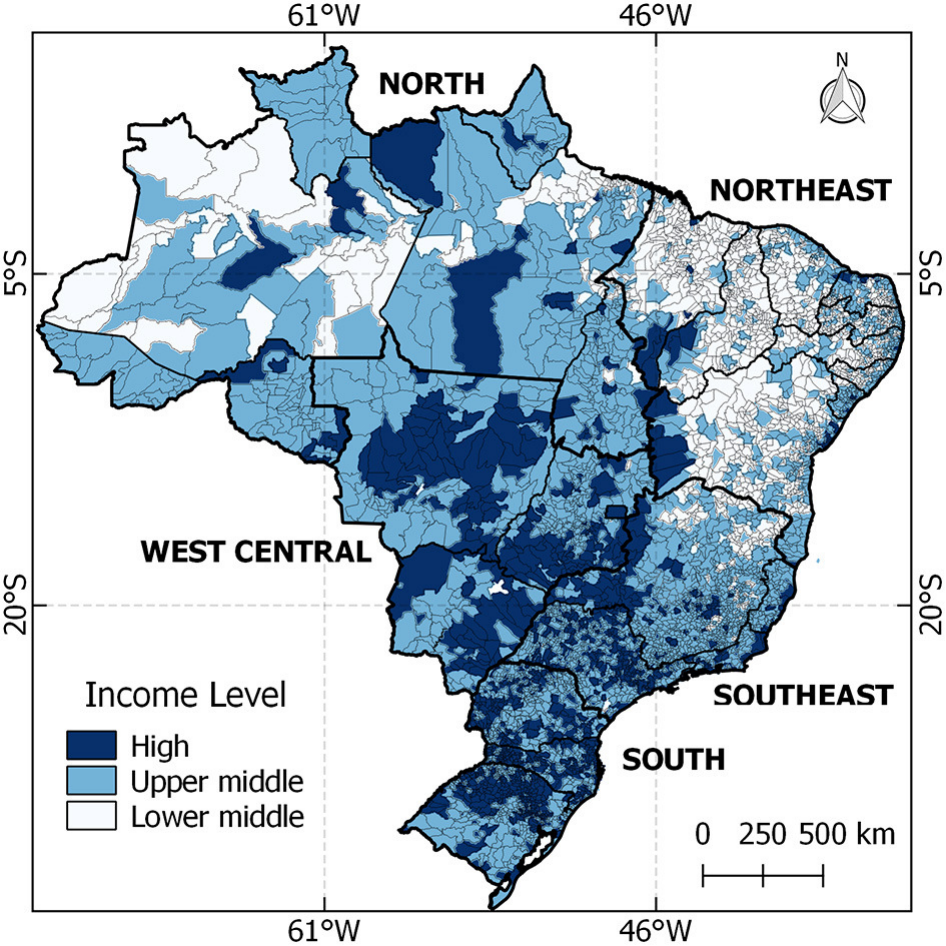
Location of Brazil and distribution of national income for each municipality.

Demographic and socioeconomic indicators from Brazil were extracted from the Brazilian Institute of Geography and Statistics (IBGE, acronym in Portuguese) (16). Brazil occupies 8,510,295 km^2^ in South America, and the estimates for the resident population in the 5,570 municipalities are 210,147,125 inhabitants. The income level of each municipality was based on the protocols used by the World Bank income classification (Figure 1) (17).

#### Health Care Facilities Infrastructure

The first step to verify the access of the population to ICU beds in Brazil was to geolocate all centers with ICU beds in Brazil. The Brazilian Health System involves public and private providers performing an extensive network of health care services (18). According to the Brazilian Society of Intensive Care (AMIB), the system comprises 8,011 health establishments, in which 1,961 have intensive care units, and 6,050 establishments can perform only low complexity care (19). Therefore, not all municipalities have high-complexity centers (HCC), especially in remote regions disconnected from the network that provides emergency care services (ECS) (18). Thus, the population that requires high-complexity health procedures frequently needs to be referred to larger municipalities (18). The list of establishments with ICU beds was retrieved from the National Register of Health Facilities (CNES acronym in Portuguese) (20), and their location was gathered using R software with the package ggmap.

#### The Recommended Number of Professionals and Equipment

To evaluate the number of professionals and equipment available, we used the recommendations of the Resolution of the Collegiate Board, created by The National Health Surveillance Agency (ANVISA, acronym in Portuguese). The ANVISA is an agency subordinate to the Ministry of Health and responsible for the quality health regulations and services of the country. The Resolution of the Collegiate Board N°7 demands a qualified multidisciplinary team in the intensive care unit, composed of (a) one intensivist for every 10 beds; (b) one nurse for every eight beds; (c) one nursing technician per bed; and (d) one physiotherapist for every 10 beds (21). In the infirmary, the recommendations are (e) one physician per 10 beds; (f) one nurse per 10 beds; (g) one nursing technician per two beds; and (h) one physiotherapist for every 10 beds (21). Lastly, two ventilators per bed are required (21).

#### Data Sources and Variables

All data on infrastructure and human resources were collected from the National Register of Health Facilities (CNES, acronym in Portuguese) (20). Information about COVID-19 cases was collected from the Secretariat of Surveillance of the Ministry of Health (22). Additional data were collected from IBGE databases (see Table 1 for all datasets) (16). All data in CNES, COVID-19, and IBGE are freely available through an open data access platform.

**Table 1:** Data source, variables, date, and data used to develop this research in Brazil, 2020.

**Source**	**Variables**	**Date range**	**Data entries**	**Reference**
COVID-19 surveillance	Number of cases; Number of deaths.	June 2020	Confirmed cases and deaths by COVID-19	(22)
CNESn National registration of health establishments	Limits of regional health entities; Number of ICU beds; Number of infirmary beds; Number of ventilators; Number of health professionals (intensivists, physicians, intensive care nurses, nurses, nursing assistants, and physiotherapists); Number of new adult and pediatric ICU beds exclusively for COVID-19.	February to June 2020	**February** 438 regional health entities 35,682 ICU beds; 426,388 infirmary beds; 65,411 ventilators; 18,716 intensivists; 564,529 Physicians; 2,768 intensive care nurses; 263,315 nurses; 710,846 nursing technicians; and 83,040 physiotherapists; **June** 17,240 new ICU beds exclusively for COVID-19.	(20)
IBGE Brazilian institute of geography and statistics	Population; Cartographic maps;	Estimate in 2019	Brazil: 210,147,125 inhabitants; North: 18,430,980; Northeast: 57,071,654; Midwest: 16,297,074; Southeast: 88,371,433; South: 29,975,984; Shapefiles for the country, states and regions.	(16)
World bank	Income level	2013	Income level for each municipality	(17)

### Data Analysis

A comparison between recommendations with the actual numbers was performed for the numbers of professionals, equipment, and the distribution of health facilities to assess the adequacy of the Brazilian HCC network. The accessibility to establishments with ICU beds was analyzed based on the two-step floating catchment area (2SFCA) (23). The accessibility index resulting from the 2SFCA was assessed to highlight regions of the country facing lack of access to ICU.

#### Distribution of ICU and Hospital Beds and Professionals

The number of health professionals and equipment was extracted from the CNES database. The results were summed and divided by the number of inhabitants into each of the five administrative regions of Brazil (North, Northeast, Midwest, Southeast, and South).

#### Resources According to Standards

The number of ICU beds and infirmary beds was divided by the number of professionals and ventilators within the same health region, aiming to assess whether the resources comply with the standards in each health region defined by ANVISA. Then, the results were plotted on choropleth maps. The maps were performed using software QGIS version 3.16.6.

#### Spatial Distribution of COVID-19 Cases and Emergency Care Accessibility

The number of cases and mortality of COVID-19 was summarized and plotted in choropleth maps. The analysis of geographical accessibility to ICU beds was performed using the 2SFCA (23). The 2SFCA creates an index of accessibility, considering the geographical area weighted by population within the region. The first step is to create a radius of 120 km surrounding each HCC to verify the number of people most likely to be covered within a traveling distance of 2 h driving at 60 km/h (18). The second step creates a ratio (index) dividing the number of ICU beds by the number of people covered within the radius. Therefore, the accessibility index refers to the availability of ICU beds per population in each municipality. For this study, two accessibility indexes were created. The first one included regular ICU beds, which verifies the accessibility to HCC in February 2020, when Brazil did not report any official COVID-19 cases. The second refers to the access to new ICU beds by June 2020, when Brazil faced a high number of critical cases. Finally, a hotspot analysis was performed using Getis-Ord Gi^*^ in ArcMap software version 10.5 to identify regions with significant clustering access to ICU beds. This analysis uses the accessibility index and the incidence of COVID-19. It is possible to verify whether the formation of clusters in regions with access to health services is located within regions with a high incidence of the disease.

### Ethical Approval

This study did not require ethical approval because no patient information was accessed, and all data are in the public domain (Resolution 510/2016 of the National Health Council).

## Results

### COVID-19 in Brazil

**Figure 2:**
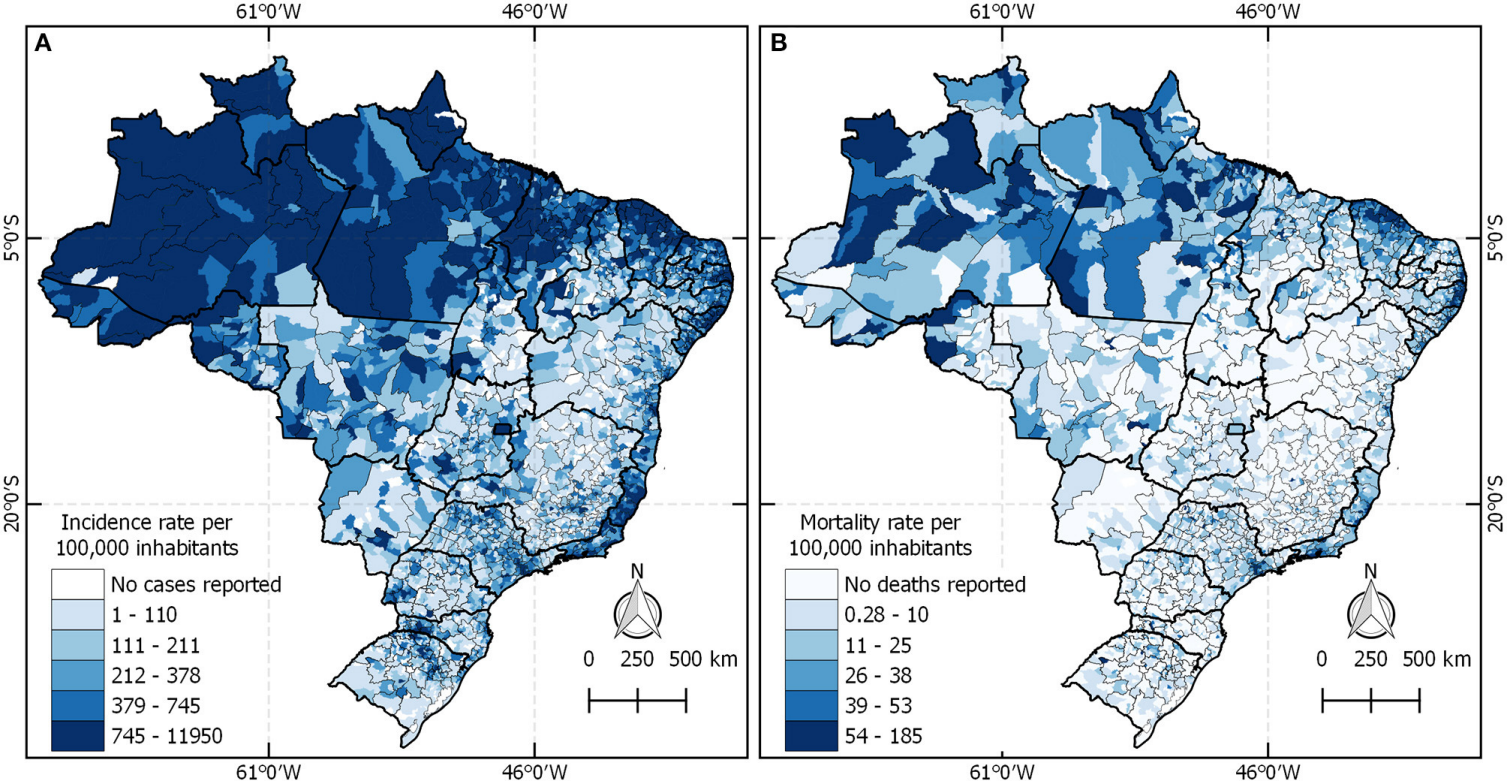
The COVID-19 incidence and mortality rate in Brazil.

Panel A in Figure 2 depicts the COVID-19 incidence by Brazilian municipality up to June 2020. The darker colors indicate higher levels of COVID-19 cases or mortality. It is possible to observe that all states and regions were presenting COVID-19 cases, especially the states of Amazonas, Amapá, Espiríto Santo, and Santa Catarina. In terms of deaths, all state capitals in the north, northeast, and southeast regions presented high levels of mortality compared with the other states in the country.

### Resources

As of February 2020, Brazil had 35,682 ICU beds, 426,388 hospital beds, and 65,411 ventilators. Additionally, Brazil has registered 18,716 intensivists, 564,529 general physicians, 2,768 intensive care nurses, 263,315 nurses, 710,846 nursing technicians, and 83,040 physiotherapists. The southeast region had the highest per population ICU beds, ventilators, physicians, nurses, and technicians, while the north region had the lowest (Table 2).

**Table 2:** Rates of different types of bed and health professionals per population in each region in Brazil^*^ (beds or health professionals/10,000 inhabitants).

**Region**	**ICU** **beds**	**Infirmary beds**	**Physicians**	**Nurses**	**Nursing** **technicians**	**Ventilators**
North	0.95	16.47	12.74	8.82	28.87	1.91
Northeast	1.19	20.00	17.60	11.62	27.39	2.19
Midwest	2.08	22.64	23.85	9.90	33.17	3.78
Southeast	2.11	19.46	34.70	14.14	38.57	3.82
South	1.70	24.33	31.72	13.21	35.46	3.13
Brazil*	1.70	20.29	26.86	12.53	33.82	3.11

* *The Brazilian Institute of Geography and Statistics (IBGE) estimates that Brazil has 210,147,125 inhabitants*.

**Figure 3:**
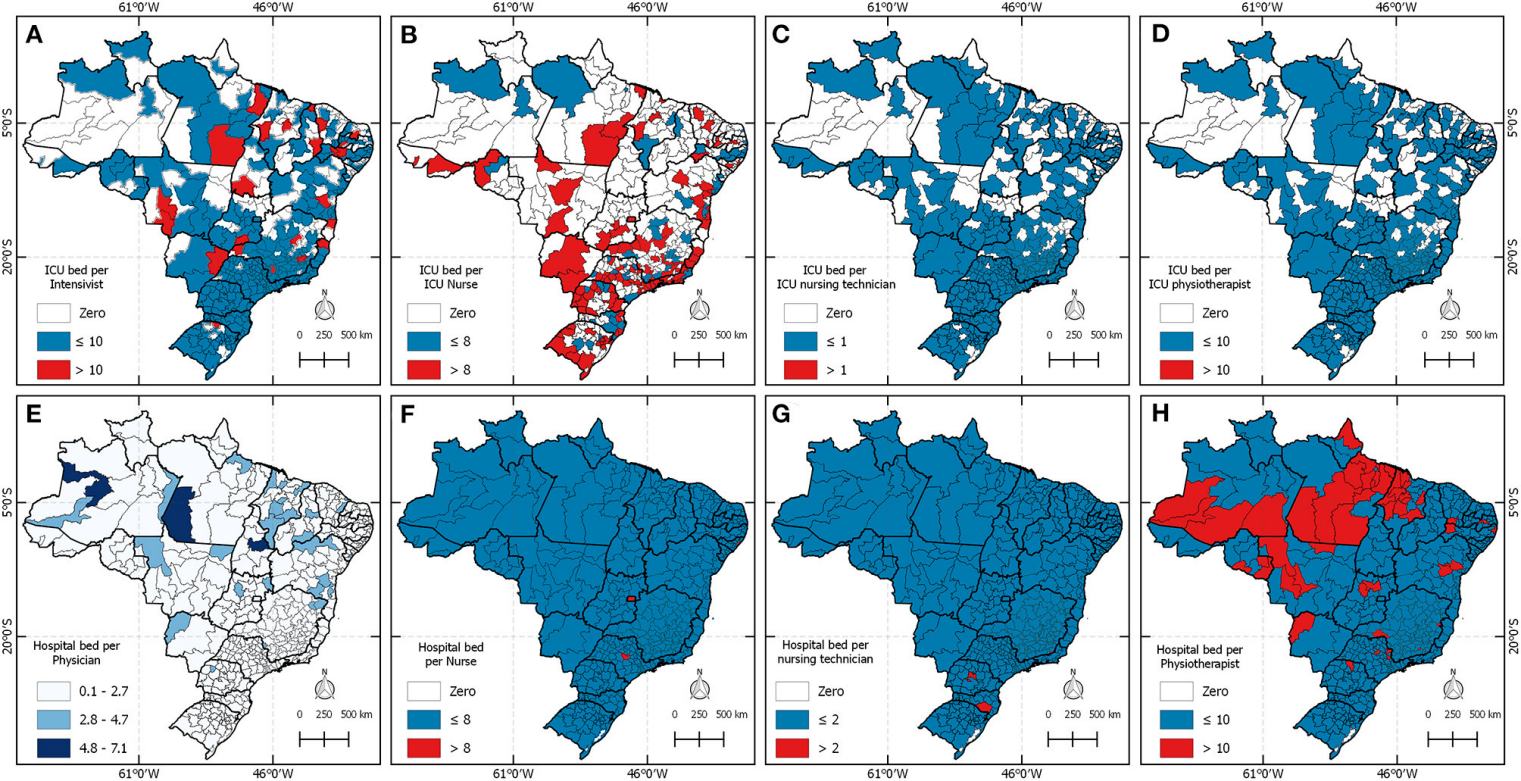
Health regions in Brazil where the conditions of beds, equipment, and professionals are demonstrated according to ANVISA recommendations.

Figure 3 shows the distribution of professionals, beds, and ventilators across the country and classifies this distribution according to the recommendations. Blue regions on the map indicate that the amount of the analyzed resource is in accordance with the norms, while red regions show that the region is in disagreement. In A, a large part of the national territory has intensivists working as recommended by ANVISA. However, 132 health regions do not have intensivists or do not have ICU beds, and 24 regions are operating beyond the recommendations. In B, only 53 regions are operating accordingly, 267 regions do not have ICU beds or intensive care nurses, and 117 are operating above the limit. Panels C and D show that most regions operate with the recommended number of nursing assistants and physiotherapists in the ICU, and have regions where there are neither such professionals nor beds. In E, all regions of the country operate with an adequate number of physicians in the infirmary. However, it is still possible to observe that nurses, nursing assistants, and physiotherapists are taking care of infirmary beds beyond the preconized.

**Figure 4:**
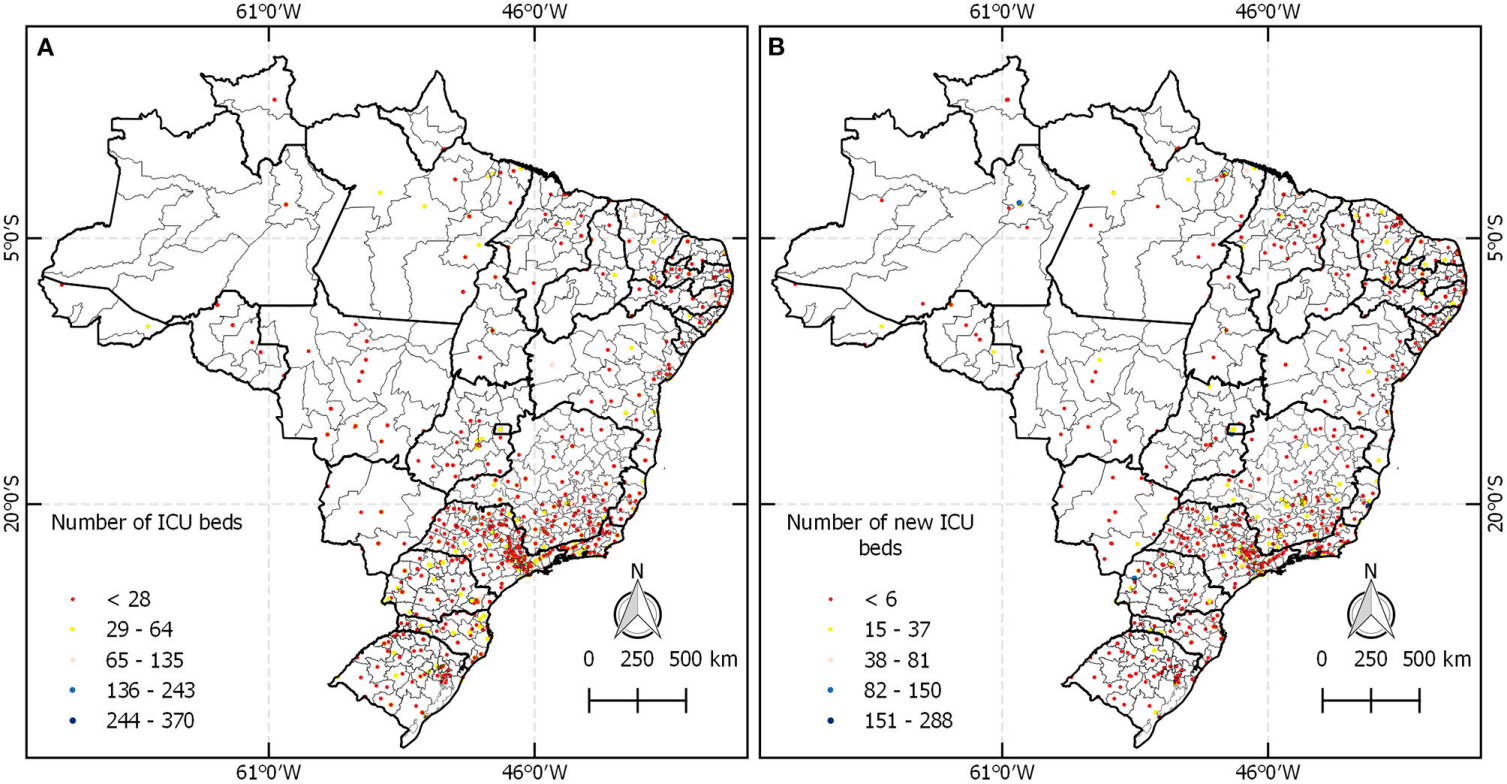
**(A)** Location of beds of regular intensive care units. **(B)** Distribution of new ICU beds.

The panel A in Figure 4 shows the distribution of all 35,682 regular intensive care beds in Brazil. Panel B shows 17,260 new ICU beds created until June 2020, where it is observed that most of the new ICU beds were located in the south and southeast regions, while the north region created many fewer.

**Figure 5:**
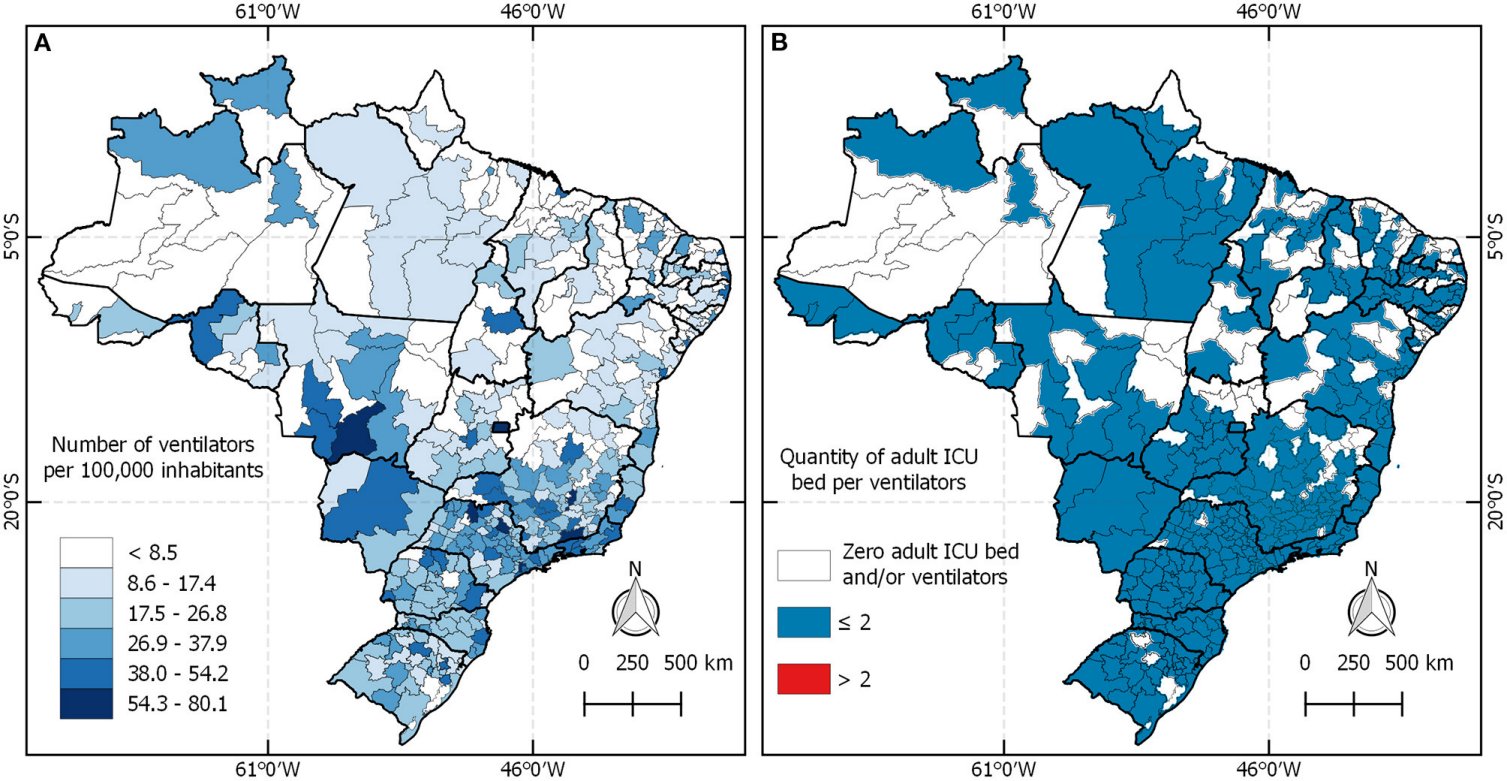
**(A)** Distribution of ventilators in Brazil for every 10,000 inhabitants. **(B)** Ventilators per bed according to ANVISA.

Figure 5 presents the distribution of ICU beds per ventilator and shows that there are ICU beds in health regions in the north without ventilators available, or there are neither ICU beds nor ventilators.

### Accessibility

**Figure 6:**
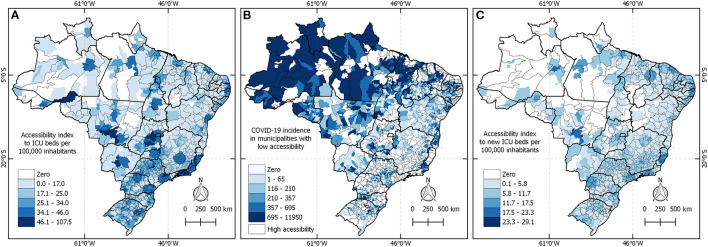
Brazilian HCC network to tackle COVID-19 in 2020. **(A)** Shows the accessibility index in February. **(B)** Presents the disease incidence in the regions that lack HCC. **(C)** Exhibits the accessibility index of new ICU beds created to tackle COVID-19.

Figure 6 presents three maps characterizing the access to HCC (establishments with ICU beds) in Brazil. Panel A shows the accessibility index to ICU beds per population and highlights a higher accessibility index in the Brazilian State capitals. Map B shows COVID-19 incidence only in municipalities where the accessibility index is below the national mean of 21.72 ICU beds per 1,000 inhabitants. Therefore, every municipality highlighted in map B has challenges in terms of HCC and high COVID-19 incidence, especially those municipalities in darker colors because they have a high incidence of COVID-19 and lack access to ICU beds. Map C presents the accessibility index to new ICU beds only, in which the results corroborate with the previous figures where the north region has lower accessibility to ICU beds in comparison to the rest of Brazil.

**Figure 7:**
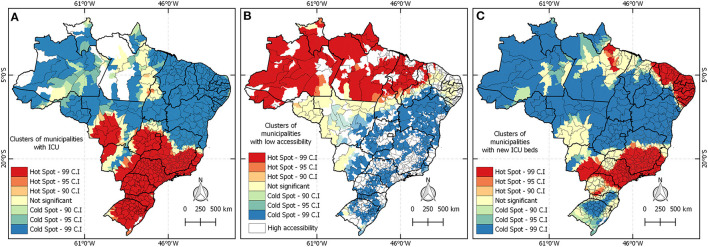
Spatial clusters of access to ICU beds dedicated to tackling COVID-19 in 2020 in Brazil. **(A)** Shows the accessibility index in February 2020. **(B)** Presents the high incidence of the disease and the lack of emergency care services. **(C)** Exhibits clusters of accessibility index of new beds created until June 2020.

Figure 7 shows the result of spatial clustering analysis based on the indexes presented in Figure 6. Panel A exhibits clusters in midwestern, southeast, and south regions. Regions covered by the red layer represent the spatially significant group of municipalities with higher access to ICU beds. On the opposite side, the blue layer highlights the regions facing barriers of accessibility. Only in B, the red color covering municipalities in the north and northeast regions indicates regions of high-incidence COVID-19 and low numbers of ICU beds. Panel C illustrates the cluster of accessibility to new ICU beds. The red hotspots on maps B and C did not overlap, meaning that the new ICUs were not created in the regions that need them most.

## Discussion

Our study showed that Brazil has significant regional disparities in infrastructure and professional resources that affect health outcomes during COVID-19 pandemic. In general, our findings demonstrated a health care system suffering from geographical inequalities (6). However, the North region was the most affected by the pandemic due to the dispersion of cases of COVID-19, as demonstrated by Castro et al. (24), and corroborated with our study showing the lack of access to hospital resources.

The oxygen crisis in Manaus can be considered as an example of the consequences of the inequity distribution of emergency resources (25). During the first wave of COVID-19, around May 2020, Amazonas also experienced an exponential increase of deaths at home that led to a collapse of both the health care and funerary systems (25). Despite the consequences associated with the first wave, Manaus decided to relax social-distancing measures. When the second COVID-19 wave struck, oxygen shortages led to the deaths of up to 40 COVID-19 ICU patients in Manaus, in January 2021 (25). Key failures at the local and national levels contributed to these tragedies.

In Brazil, health professionals are not evenly distributed geographically to accomplish the Collegiate Board Recommendations Resolution, especially those who work in ICU. In terms of nursing and physicians in ICU, many regions have few critical care workers. These professionals care for more beds than recommended, potentially putting patients at risk of adverse outcomes (26). Kerlin et al. (27) verified that overworked professionals in critical care are more likely to experience burnout and provide unsatisfactory quality of care, which may have significant consequences for patient care and the healthcare system. In addition, the intense and emotionally charged work drains healthcare providers physically and emotionally to the point where they may get sick, further limiting their ability to treat patients and reducing the available workforce (28). Therefore, Brazilian regions with overworked professionals may face a more significant challenge against COVID-19.

Health resources in Brazil are majorly accessible to the people living in urban areas, with abundant access focused in larger centers in the southeast and south regions such as São Paulo, Rio de Janeiro, Paraná, and Santa Catarina States. On the other hand, underdeveloped, rural, and sparsely populated regions did not receive the same attention in comparison to the developed areas. The uneven distribution of health resources led to a disproportionate number of cases and mortality of COVID-19.

Here we analyzed the distribution of ICU beds in February, before the arrival of COVID-19 in Brazil, to verify the preparation for health resources management. Initially, few hospital beds were relocated to serve only patients with COVID-19, which reduced the number of beds available for patients with other pathologies. This maneuver affected negatively densely populated regions where they usually have a greater availability of beds. However, the impact was even worse in regions with few beds. Therefore, to circumvent this situation, new ICU beds were created to serve patients with COVID-19, apart from preexisting ones. But when the first wave hit Brazil by June, we verified that new beds were mainly added in the south and southeast regions, where there were already higher numbers of beds in comparison to other regions. Thus, those regions with few beds faced a disproportional challenge to overcome COVID-19.

In Brazil, existing socioeconomic inequalities have affected the course of COVID-19, with a disproportionate adverse burden on states and municipalities with high socioeconomic vulnerability (29). The results we got are aligned with other studies in the literature, emphasizing that the distribution of emergency resources dedicated to tackle COVID-19 followed the patterns of inequalities present in the country before the pandemic (29). Part of this outcome was due to a lack of central coordination to better drive the opening of new ICU beds, distribution of ventilators, as well as the other resources to support emergency care. The political context posed a major challenge to the response. The risk of the virus has been downplayed by the current political leadership in Brazil, and messages from the federal government have been mixed with regard to physical distancing restrictions, use of face masks, and reopening plans, among other aspects (29).

The surge of critical supply shortages affected all countries and led to a worldwide race for ventilators (30). The availability of ventilators in Brazil was not satisfactory in several regions, especially in the north and northeast regions. Therefore, the most severe cases of COVID-19 may be more overwhelming in these regions (31). This situation predicted and stated that Brazil faced a double crisis: COVID-19 and internal political conflicts. The federal government went against the Ministry of Health and WHO's technical recommendations when COVID-19 reached Brazil (32). Thus, each administrative level implemented isolated responses to prepare for COVID-19 with limited country-level coordination. The effective absence of a Brazilian government response to COVID-19 made the country particularly susceptible to outcome inequities (33). Furthermore, healthcare has been overlooked by the government, delaying and promoting insufficient resources to aid the most vulnerable population (34).

This study presents its strengths. First, all data are publicly available. In addition, this is one of the first studies to perform spatial analysis to verify the uneven distribution of ICU beds in Brazil. However, the study also presents some limitations. Biases are commonly found in observational studies based on secondary data and cannot be ruled out, especially here since the data were obtained from the government. The accessibility index to ICU beds was performed, taking into consideration that everyone within the radius of 120 km from HCC had the same chance to be treated. However, the geography in Brazil is highly diverse, and the population in the Amazon region probably had higher challenges since their transportation frequently relies on rivers. Furthermore, we could be overestimating the accessibility of large centers because patients deliberately travel great distances to larger centers to have a greater chance of being assisted, overloading this center as it would be serving a population outside the scope stipulated by the analysis.

In conclusion, Brazil has a myriad of infrastructure challenges limiting its ability to treat critically ill patients. We have demonstrated an insufficient number of ICU beds and ventilators, and a severely limited number and inequitable healthcare professional distribution. Additionally, the allocation of new beds did not address areas with known gaps in care and health infrastructure accessibility. Therefore, the SARS-CoV-2 epidemic in Brazil may be devastating, especially in areas with limited health infrastructure. Lastly, this study suggests that strong leadership is needed to coordinate the response efforts against COVID-19.

## Data Availability Statement

The raw data supporting the conclusions of this article will be made available by the authors, without undue reservation.

## Author Contributions

LS contributed to the investigation, software, formal analysis, and writing—original draft. AC, PI, and GR made substantial contributions to the data curation and formal analysis. LA, IP, JC, PV, and TH performed the methodology, formal analysis, validation, and visualization. CS contributed to the supervision and writing—review and editing. JN and RF contributed equally to the conceptualization, project administration, and contributed to the supervision and writing—review and editing. All authors contributed to the article and approved the submitted version.

## Conflict of Interest

The authors declare that the research was conducted in the absence of any commercial or financial relationships that could be construed as a potential conflict of interest.

## Publisher's Note

All claims expressed in this article are solely those of the authors and do not necessarily represent those of their affiliated organizations, or those of the publisher, the editors and the reviewers. Any product that may be evaluated in this article, or claim that may be made by its manufacturer, is not guaranteed or endorsed by the publisher.
